# Ecological Study of HIV Infection and Hypertension in Sub-Saharan Africa: Is There a Double Burden of Disease?

**DOI:** 10.1371/journal.pone.0166375

**Published:** 2016-11-17

**Authors:** C. Angkurawaranon, D Nitsch, N Larke, A. M. Rehman, L. Smeeth, J. Addo

**Affiliations:** 1 Department of Family Medicine, Faculty of Medicine, Chiang Mai University, Chiang Mai, Thailand; 2 Department of Non-Communicable Disease Epidemiology, Faculty of Epidemiology and Population Health, London School of Hygiene and Tropical Medicine, London, United Kingdom; 3 Medical Research Council Tropical Epidemiology Group, Department of Infectious Disease Epidemiology, Faculty of Epidemiology and Population Health, London School of Hygiene and Tropical Medicine, London, United Kingdom; UCL Institute of Child Health, University College London, UNITED KINGDOM

## Abstract

An ecological correlation study of the prevalence of hypertension with human immunodeficiency virus (HIV) prevalence in sub-Saharan Africa was conducted to determine the extent to which these conditions coincide at country level.

**Methods:**

Data on prevalence of hypertension were derived from a systematic search of literature published between 1975 and 2014 with corresponding national estimates on HIV prevalence and antiretroviral therapy (ART) coverage from the Demographic and Health Surveys and the joint United Nations Programme on HIV/AIDS databases. National estimates on gross national income (GNI) and under-five mortality were obtained from the World Bank database. Linear regression analyses using robust standard errors (allowing for clustering at country level) were carried out for associations of age-standardised hypertension prevalence ratios (standardized to rural Uganda’s hypertension prevalence data) with HIV prevalence, adjusted for national indicators, year of study and sex of the study population.

**Results:**

In total, 140 estimates of prevalence of hypertension representing 25 nations were sex-and area-matched with corresponding HIV prevalence. A two-fold increase in HIV prevalence was associated with a 9.29% increase in age, sex and study year-adjusted prevalence ratio for hypertension (95% CI 2.0 to 16.5, p = 0.01), which increased to 16.3% (95% CI 9.3 to 21.1) after adjusting for under-five mortality, GNI per capita and ART coverage.

**Conclusions:**

Countries with a pronounced burden of HIV may also have an increased burden of non-communicable diseases such as hypertension with potential economic and health systems implications.

## Introduction

Sub-Saharan Africa (SSA) has been greatly affected by the human immunodeficiency virus (HIV) and Acquired Immunodeficiency Syndrome (AIDs) epidemic. An estimated 68% (23, 200 000) of all people infected with HIV globally were reported by the World Health Organization (WHO) to be residing in the region in 2010.[1] At the same time, there is evidence of an increasing burden of non-communicable diseases (NCDs) occurring in SSA resulting in a double-burden of NCDs and communicable diseases in a region with the least financial as well as human resources to deal with this effectively.[2–4] Higher rates of NCDs such as stroke have been reported from SSA, particularly urban areas, compared to higher income countries possibly due to undiagnosed and poorly treated hypertension.[5] A total of 75 million people were estimated to have hypertension in SSA in 2008 and this is projected to increase to 125 million by 2025.[6]

The link between NCDs and communicable disease in SSA is being explored and some previous studies have reported associations between cardiovascular disease and risk factors such as hypertension with human immunodeficiency virus (HIV) as well as antiretroviral therapy (ART).[7–9]

Despite the observed burden of these NCDs in SSA, a large donor response has been towards communicable disease with some African countries spending a significant proportion of their health budgets on HIV/AIDS while giving less priority to funding for NCDs.[10–13] Yet, integrating lessons learned from the global response to HIV/AIDS into NCD programs may be more effective in addressing the increasing burden of NCDs in SSA.[14] An initial step to dealing effectively with the growing double burden of NCDs and communicable diseases and the associations between these, is to determine the extent to which these conditions co-exist at country level.

We therefore conducted an ecological correlation study of the prevalence of hypertension with HIV distribution in SSA. We considered hypertension because it is one of the most prevalent risk factors for cardiovascular disease in SSA. [15, 16]

## Methods

A systematic search was conducted of the MEDLINE (PubMed) electronic database, to identify studies that reported blood pressure (BP) and/or hypertension prevalence in SSA between January 1975 and December 2014. Additional searches using the Behavioral Risk Factor Surveillance System (BRFSS) and the World Health Organization (WHO) databases were conducted along with a manual search of references cited in the identified articles. The search strategy used key terms including “hypertension”, “blood pressure”, “Africa” and “Africa South of the Sahara” (“S1 Appendix”). No language restrictions were applied. Potentially relevant full articles were obtained after scanning through the retrieved titles and abstracts. The review followed the PRISMA guidelines for reporting systematic reviews.[17] The eligibility criteria for inclusion of studies were: (1) population-based studies involving black participants living in SSA and that included 400 or more participants aged 15 years and above; (2) random sampling of a defined population described or studies involving entire populations; (3) standard description of methods used in measuring blood pressure stated; (4) reporting data on the prevalence of hypertension (crude or age-adjusted) with hypertension defined as blood pressure ≥140/90mmHg or self-reported use of antihypertensive medication (Joint National Committee on Prevention, Detection, Evaluation and Treatment’s report (JNC 7 criteria)).[18] Multiple papers from the same study were grouped together and compared for consistency.

The inclusion criteria for Corresponding national estimates on HIV prevalence were obtained using the Demographic and Health Survey (DHS) database and the joint United Nations Programme on HIV/AIDS (UNAIDS) database. As access to care and socioeconomic development are also important factors towards understanding the relationship between hypertension and HIV prevalence, the national indicators for these factors were also obtained using the World Bank database.

### Variables of Interests

#### Blood pressure and hypertension prevalence

We identified studies reporting the mean systolic BP and/or prevalence of hypertension in SSA. The year of publication, year of fieldwork, area of fieldwork (whether urban and/or rural), mean age/age range and sex of the study population were extracted.

#### HIV prevalence and coverage of antiretroviral therapy (ART)

Using DHS and UNAIDS database, corresponding national HIV prevalence estimates were matched to within five years of the field work or date of publication of the BP and hypertension prevalence estimate. When possible, the national HIV prevalence estimates were matched according to area of study (urban/rural) and sex of the study population based on the hypertension prevalence estimates. For ART coverage among adults, the estimates were sex matched to within five years of the national HIV prevalence estimates“S1 Table”. HIV prevalence and ART coverage were positively skewed, thus a logarithm transformation (base 2) was done, with a one-unit change translating to a doubling.

#### Other variables of interests

Under-five Mortality was considered as a proxy variable for improved social determinants and vaccine roll-out. Improved social determinants may be a confounding variable of the associations seen in this study. The under-5 mortality rate (per 1,000 live births) was obtained from the World bank’s database. The closest estimates, within 5 years from year of fieldwork in the BP/hypertension prevalence estimates. Where this was not possible, the closest estimates within 5 years of HIV prevalence estimates were used.

Gross National Income (GNI) per capita was used as a measure of socioeconomic development. For most countries, this indicator was matched with the year of fieldwork in the BP/hypertension estimates. Where this was not possible, it was matched to the year of HIV prevalence estimates. In the few countries where GNI per capita was not reported annually, it was matched to within 5 years of the fieldwork for the BP/hypertension estimates or within 5 years of HIV prevalence estimates “S1 Table”.

### Analyses

Stata version 13 was used for all analyses. The main exposure was log-2 transformed HIV prevalence. The main outcome of interest was age-standardized prevalence ratios of hypertension. Results on mean BP are provided in the supplementary file “S2 Table”.

Age-standardized hypertension prevalence ratios were calculated using indirect standardization methods. The standard age-specific hypertension prevalence came from a study conducted in rural Uganda [19]. This rate was chosen as it was considered the most reliable age-specific estimates from our literature search. Using the age range and year of fieldwork from each hypertension study, the standard rate and each study’s crude estimate of hypertension prevalence were applied to that country’s age distribution. The country’s age distribution (matched to within five years from the year of fieldwork) was obtained from United Nations, Population Estimates and Projection Section database.

Pearson correlation and Spearman correlation were used to test the association between log-transformed HIV prevalence with age-standardized prevalence for hypertension and mean blood pressure. These associations were further investigated by stratifying on area and sex of the study population. Age-standardized prevalence ratios for hypertension were normally distributed (as evidenced by residuals, data not shown) and modeled using linear regression with robust standard errors to allow for clustering at country level, as well as adjusting for year of HIV estimate (in 5 year bands). When analyzing the association between log-transformed HIV prevalence and mean systolic BP linear regression was used controlling for mean age of the study population, using robust standard errors to allow for clustering at country level, as well as adjusting for year of HIV estimate (in 5 year bands). Under-five mortality and GNI per capita and log-transformed ART coverage, were entered in subsequent models. Sequential models were used to evaluate confounding, with model 1 representing crude results, model 2 investigating how age and sex and year of study confound the associations, model 3 was additionally adjusted for under 5 mortality and GNI per capita as socio-economic confounders. Lastly model 4 adjusted in addition to previous variables for log (base 2) ART coverage, to better understand the additional association that may be mediated by this potential pathway variable on the association between HIV prevalence with age-standardized prevalence ratio for hypertension and mean systolic blood pressure.

## Results

**Fig 1:**
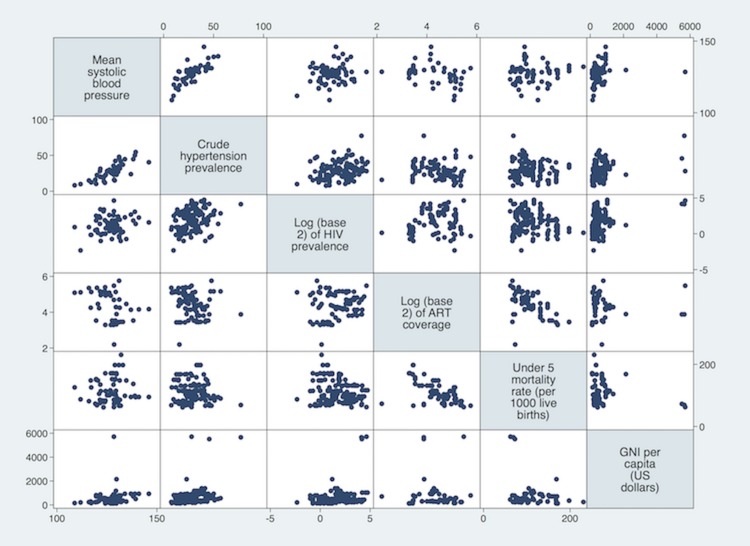
Scatter plots of crude associations between Mean Blood Pressure, Crude Prevalence of HT, Log transformed Prevalence of HIV, Under-five Morality and GNI per capita.

The systematic review of hypertension studies found 75 eligible research articles. Two articles were excluded as one reported mean blood pressure by ethnicity (but not urban/rural location) [20] and another on the exposure to famine (but not reporting the required blood pressure results for use in this ecological study)[21].The data used for this study are available as supporting information“S1 Table”. In total, combined with the estimates from the BRFSS and WHO database, we found 221 estimates of mean systolic BP and/or prevalence of hypertension from 28 nations in SSA conducted between 1975 and 2014. Of these estimates, 140 (59.4%) representing 25 nations, were matched with corresponding HIV prevalence according to area and sex of the study population. It is these sex-and area-matched estimates between BP/hypertension prevalence and HIV that are used in further analysis. From the 140 sex-and-area matched estimates, 65 reported values on mean BP and 123 reported values on hypertension prevalence (48 reported both blood pressure and hypertension prevalence) “S1 Fig”. The crude relationship between mean BP, crude hypertension prevalence, log-transformed HIV prevalence and other variable of interests are displayed in “Fig 1”.

### Association between Prevalence of HIV and Age-Standardized Prevalence of Hypertension

**Fig 2:**
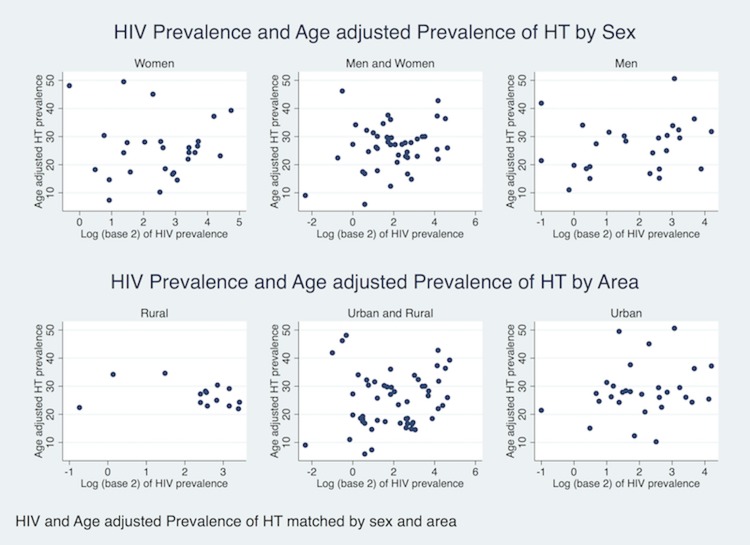
Association between Prevalence of HIV and Mean Systolic Blood Pressure by sex and area.

There was a positive association between the log-transformed prevalence of HIV and crude prevalence of hypertension (Spearman’s rho = 0.26, p<0.001). This association attenuated after indirect standardization for age and lost statistical significance. (Spearman’s rho = 0.16, p = 0.16). Stratifying the association according to sex of study population did not materially alter the association but there was some evidence that urban/rural area of study may modify the association between prevalence of HIV and age-standardized prevalence of hypertension (“Fig 2”). However, these crude data were not taking account of clustering at country level and year of study. (Results for stratified analysis by sex were area-specific and results for stratified analysis were gender specific.) Subsequent linear regression modeling taking account of country clustering of data showed that every doubling of HIV prevalence was crudely associated with an 6.95% increase in age-standardized prevalence ratio for hypertension (95% CI -0.03 to 19.9%, p = 0.05). When adjusting the association further for sex and study year, a doubling of HIV prevalence was associated with a 9.3% increase in age-adjusted prevalence ratio for hypertension (95% CI 2.0 to 16.5%, p = 0.01). When further adjusting for under-five mortality and GNI per capita, a two-fold increase in HIV prevalence was associated with an 14.1% increase in age-adjusted prevalence ratio for hypertension (95% CI 6.6 to 21.5%, p<0.01). This association remained strong despite adjusting for ART coverage, with a doubling in HIV prevalence being associated with a 16.3% increase in age-adjusted prevalence ratio for hypertension (95%CI 9.3 to 21.1%, p<0.01). Under five mortality was in all analyses strongly negatively associated with age-standardised prevalence ratio of hypertension, whilst GNI per capita showed only weak associations at country level. In crude analyses a doubling of ART coverage was negatively associated with age-standardised prevalence ratio of hypertension, which attenuated and reversed sign after taking account of confounding by the other variables in the model “Table 1”.

**Table 1:** Association between HIV prevalence with Age-Standardized Prevalence Ratios for Hypertension.

	Age-Standardized Prevalence Ratio for Hypertension (% increase)			
	β* (95% CI)	β* (95% CI)	β* (95% CI)	β* (95% CI)
Variable (unit)	Model 1^#^	Model 2^##^ N = 97	Model 3^###^ N = 96	Model 4^###^ N = 65
Log (base 2)prevalence of HIV	• 6.95 • (-0.03 to 13.9) • p = 0.05	• 9.29 • (2.02 to 16.5) • p = 0.01	• 14.1 • (6.60 to 21.5) • p<0.01	• 16.3 • (9.34 to 21.1) • p<0.01
Under five mortality(per 10 live birth)	• -31.6 • (-55.1 to 2.93) • p = 0.07	—	• -118.6 • (-162.1 to -74.9) • p<0.01	• -84.3 • (-143.0 to -25.6) • p<0.01
GNI per capita(in 100 US dollars)	• 0.90 • (-0.10 to 1.89) • p = 0.08	—	• -0.63 • (-2.59 to 1.33) • p = 0.53	• -0.63 • (-2.61 to 1.35) • p = 0.54
log(base2) ARTcoverage	• -11.8 • (-29.1 to 5.43) • p = 0.18	—	—	• 3.23 • (-14.8 to 21.2) • p = 0.73

* β represent changes in age-standardized prevalence ratio for hypertension per unit increase in co-variable of interest

^#^ Model 1 values are unadjusted estimates (represent separate statistical models)

^##^ Model2 value is adjusted for age and sex of the study population and year of HIV estimate (in 5 year bands)

^## #^Model 3 and 4 values are adjusted for all co-variables included in the same statistical model in the same column and sex of study population and year of HIV estimate (in 5 year bands)

GNI = Gross National Income; ART = Antiretrovial therapy; All models used robust standard errors to allowing for clustering by countries

### Association between Prevalence of HIV and Mean Systolic Blood Pressure

**Fig 3:**
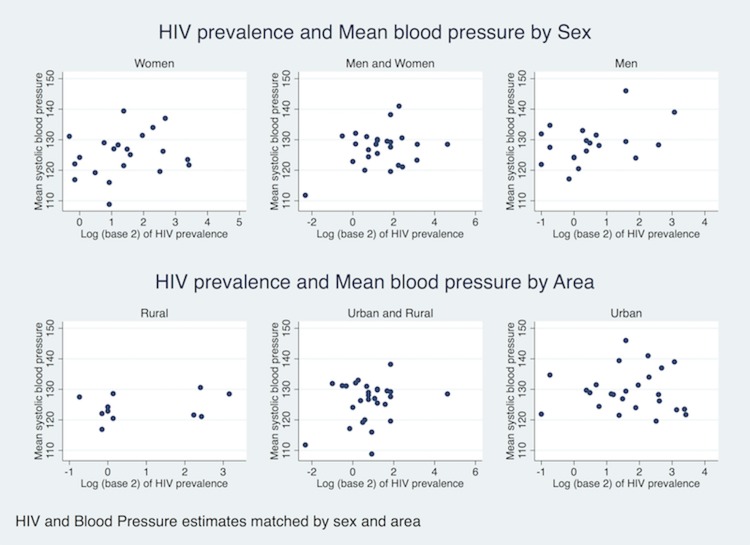
Association between Prevalence of HIV and Age-standardised Prevalence of hypertension by sex and area.

There was some weak evidence for a positive association between log-transformed HIV prevalence and mean BP (Pearson correlation = 0.15, p = 0.11). Stratifying on sex and urban/rural area of study did not materially change the association “Fig 3”. Using linear regression (using robust standard errors for clustering at country level), every doubling in HIV prevalence was associated with 0.9 mmHg increase in mean systolic BP (95% CI -0.43 to 2.23, p = 0.18). Further adjustments for age, sex, year of study population, under five mortality, GNI per capita and ART coverage increased the effect size for every double of HIV prevalence to 1.24 mmHg (95% CI -0.50 to 2.97, p = 0.16). “S2 Table”

## Discussion

This ecological study found some evidence for geographical association between HIV and hypertension, with countries with higher HIV prevalence having higher hypertension prevalence when adjusted for age, sex, year of study, markers of socioeconomic development (GNI per capita, and under-five mortality), and ART coverage.

These findings applying at country level and not to individuals suggest that countries with a pronounced burden of HIV may also have an increased population need for hypertension treatment to prevent associated NCDs. This highlights a double burden of disease with NCDs co-existing alongside infectious diseases including HIV and the treatment with ART also associated with an increased risk of cardiovascular disease.[22] The resulting double-burden of disease has significant population health, health systems and economic implications for resource limited countries in SSA.[23]

There is ongoing research on how HIV and indeed long term ART use among HIV infected individuals are associated with NCDs.[22, 24–26] HIV infection has been shown in previous studies to be associated with vascular disease and hypertension.[22, 27] Previous studies examining the association between HIV and hypertension have reported conflicting results. Whereas higher rates of hypertension have been reported among HIV-infected adults in some studies [8, 28], other studies have shown no such association.[29, 30] Traditional risk factors of cardiovascular disease (CVD) as well as increased pre-clinical atherosclerosis are possible explanations for the higher risk of CVD in HIV-infected people.[31, 32] Increased blood pressure in HIV-infected individuals have been shown to be associated with established risk factors for hypertension such as older age and higher body mass index.[33] With the increased life expectancy associated with the introduction of ART, HIV infection has become a chronic condition and risk factors for CVD associated with ageing and lifestyle are likely to play an increasingly important role in the aetiology of NCDs in HIV-infected people. Considering their shared factors, efforts to combine prevention and treatment strategies for HIV and NCDs are undoubtedly needed to address the double burden of NCD and infectious disease in SSA. Despite the observed coexistence of NCDs and infectious diseases in populations such as SSA, suggesting the need for a combined strategy in surveillance, prevention and disease control, experts, institutions and policies that support prevention and control often have limited interaction and alignment in these settings.[34] In the absence of an integrated model of care and policies addressing both HIV and NCDs effectively, morbidity and mortality from NCDs may set back or even reverse the health gains achieved in HIV-infected populations with the widespread HIV prevention and treatment efforts and the advent of ART.[35] Integrating NCD and HIV programs however depend not only on policy makers but also on relevant stakeholders including donors being willing to fund such programs.[36] Funding organizations have to consider and accept that there are areas in SSA that suffer from a wide range of health problems at the same time including both infectious diseases and NCDs. The availability of health systems that meet the multiple health needs of the population will undoubtedly improve access to care and the longer term health outcomes in areas with a double burden of infectious disease and NCDs. An effective response will additionally benefit from multi-sectoral policies and actions for dealing with disease related risk behaviours and environmental factors.[36]

Ecological studies provide a quick and cheap way of determining associations between factors of interest and outcomes but the inability to characterize potential confounders makes it difficult to draw definitive conclusions and cannot determine causality. Hence, this study had some limitations. There were some SSA countries with no data at all thus limiting generalizability. Data for hypertension were taken from several studies that had been conducted using different methodologies and involving populations with characteristics different from those used for determining HIV prevalence. Although the study tried to control for possible confounders such as age, sex and area of study, there is still potential for residual confounding. Because we were concerned that the mean blood pressure estimates are affected by a skewed country distribution in the older age range, we repeated our analyses using indirect standardization for age and report these as our main results. Our age standardized prevalence of hypertension relied on the assumption that the age distribution of each study population was the same as the national distribution. The search for hypertension studies was limited to only one (Medline) database with the possibility of missing some eligible studies. Despite these limitations, the current ecological study adds to the literature demonstrating a convergence of NCDs and infectious diseases in SSA and highlights the need for further research to better understand pathways of how communicable and non-communicable diseases interact in such populations affected by both health problems. Further research is also needed to identify innovative ways of integrating the prevention and care strategies of both NCDs and HIV in different populations in SSA and the cost effective ways of implementing policies to address these.

This ecological study does not prove or refute a direct link between HIV infection and hypertension. Indeed, more research is needed to better understand pathways of how communicable and non-communicable diseases interact in the populations who are affected by both health problems. The findings however suggest that countries with a pronounced burden of HIV may also have an increased burden of non-communicable diseases such as hypertension with potential population health, economic and health systems implications.

## Supporting Information

S1 Appendix Search strategy and terms. (PDF)

S1 Fig Flow chart of the review process. (TIFF)

S1 Table Studies and estimates used for analysis. (XLS)

S2 Table Association between HIV prevalence with Mean Systolic Blood Pressure. (DOCX)
