# Supplementary material for: Ecological Study of HIV Infection and Hypertension in Sub-Saharan Africa: Is There a Double Burden of Disease?
Source: PLoS One. 2016 Nov 17;11(11):e0166375. doi: 10.1371/journal.pone.0166375 (PMC5113946; doi:10.1371/journal.pone.0166375)
Supplement: S1 Appendix — (PDF) [file pone.0166375.s001.pdf]

Ovid Medline Search strategy

1 exp hypertension/

2 exp blood pressure/

3 ((elevat$ or high$ or rais$) adj 3 (diastolic or systolic or arterial or blood) adj pressure).tw.

4 bloodpressure.tw.

5 or/1-4

6 (Africa or Algeria or Angola or Benin or Botswana or "Burkina Faso" or Burundi or Cameroon or "Canary Islands" or "Cape Verde" or "Central African Republic" or Chad or Comoros or Congo or "Democratic Republic of Congo" or Djibouti or Egypt or "Equatorial Guinea" or Eritrea or Ethiopia or Gabon or Gambia or Ghana or Guinea or "Guinea Bissau" or "Ivory Coast" or "Cote d'Ivoire" or Jamahiriya or Kenya or Lesotho or Liberia or Libya or Madagascar or Malawi or Mali or Mauritania or Mauritius or Mayotte or Morocco or Mozambique or Namibia or Niger or Nigeria or Principe or Reunion or Rwanda or "Sao Tome" or Senegal or Seychelles or "Sierra Leone" or Somalia or "South Africa" or "St Helena" or Sudan or Swaziland or Tanzania or Togo or Tunisia or Uganda or "Western Sahara" or Zaire or Zambia or Zimbabwe or "Central Africa" or "Central African" or "West Africa" or "West African" or "Western Africa" or "Western African" or "East Africa" or "East African" or "Eastern Africa" or "Eastern African" or "South African" or "Southern Africa" or "Southern African" or "sub Saharan Africa" or "sub Saharan African" or "subSaharan Africa" or "subSaharan African").

7 5 and 6

8 limit 7 to yr="1975 - 2014"

9 (addresses or autobiography or bibliography or biography or dictionary or directory or editorial or government publications or letter or health education or qualitative or intervention or news).pt

8 not 9 (4881 papers retrieved)
