# Supplementary figures and images for: Ecological Study of HIV Infection and Hypertension in Sub-Saharan Africa: Is There a Double Burden of Disease?
Source: PLoS One. 2016 Nov 17;11(11):e0166375. doi: 10.1371/journal.pone.0166375 (PMC5113946; doi:10.1371/journal.pone.0166375)

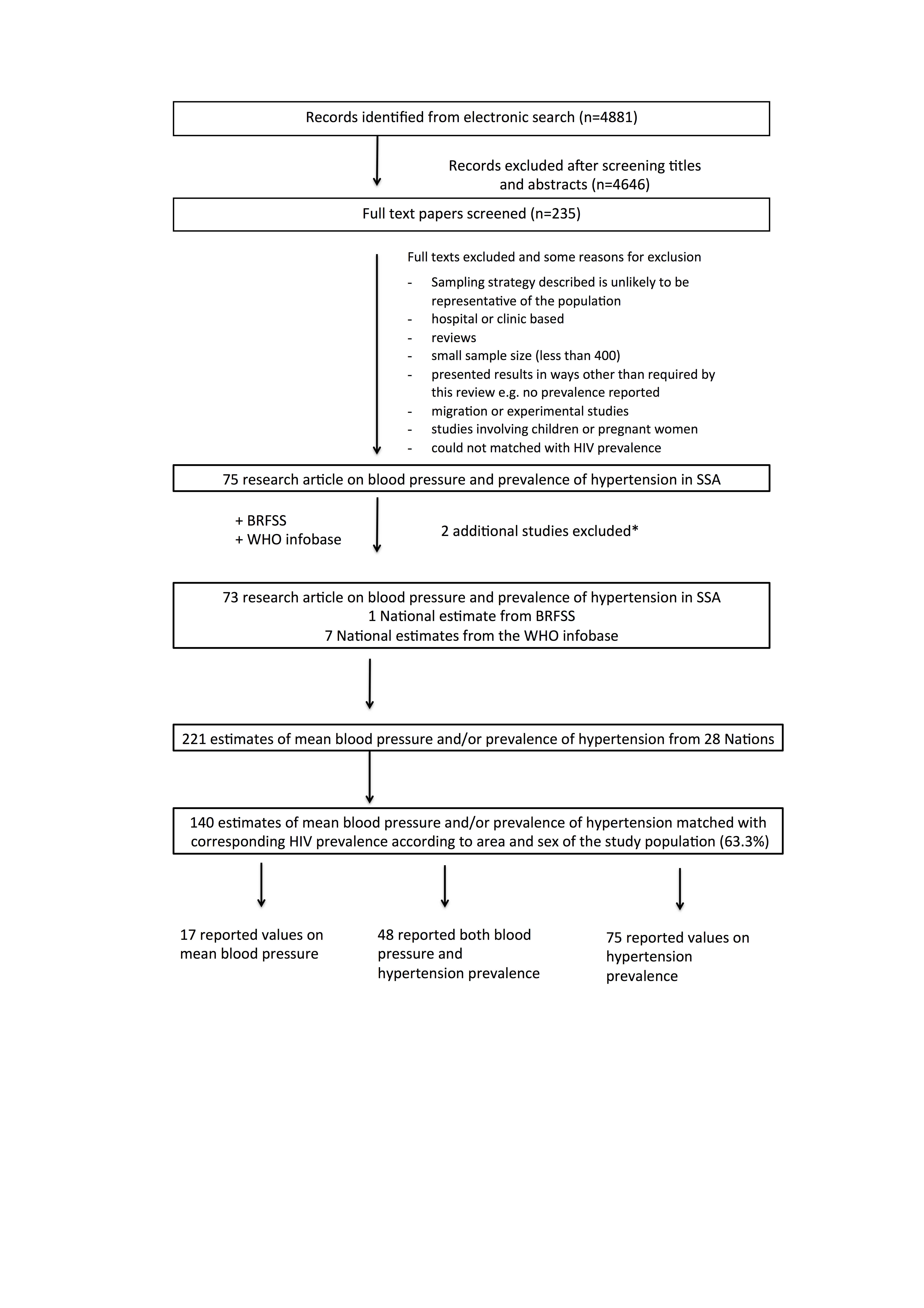

Supplement: S1 Fig — (TIFF) [file pone.0166375.s002.tiff]
