# Supplementary material for: Ecological Study of HIV Infection and Hypertension in Sub-Saharan Africa: Is There a Double Burden of Disease?
Source: PLoS One. 2016 Nov 17;11(11):e0166375. doi: 10.1371/journal.pone.0166375 (PMC5113946; doi:10.1371/journal.pone.0166375)
Supplement: S2 Table — (DOCX) [file pone.0166375.s004.docx]

**Supplementary 3 Table 2. Association between HIV prevalence with Mean Systolic Blood Pressure**

|  | **Mean Systolic Blood Pressure** | | | |
| --- | --- | --- | --- | --- |
|  | **β (95% CI)** | **β (95% CI)** | **β (95% CI)** | **β (95% CI)** |
|  | **Model 1^#^** | **Model 2^##^**  **n=48** | **Model 3^###^**  **~~n=48~~** | **Model 4^###^**  **n=29** |
| **Variable (unit)** |  |  |  |  |
| Log (base 2) prevalence of HIV | 0.90  (-0.43 to 2.23)  p=0.18 | 0.82  (-0.67 to 2.13)  p=0.28 | 0.89  (-0.55 to 2.31)  p=0.22 | 1.24  (-0.50 to 2.97)  p=0.16 |
| Mean age (years) | 0.45  (0.19 to 0.70)  p<0.01 | 0.40  (0.17 to 0.63)  p<0.01 | 0.42  (0.18 to 0.65)  p<0.01 | 0.58  (0.30 to 0.88)  <0.01 |
| Under five mortality (per 10 live birth) | -1.07  (-6.06 to 3.92)  p=0.67 | -- | 2.33  (-2.58 to 7.24)  p=0.35 | 1.26  (-6.70 to 9.22)  p=0.76 |
| GNI per capita  (in 100 US dollars) | 0.12  (-0.11 to 0.34)  p=0.30 | -- | 0.36  (-0.18 to 0.91)  p=0.19 | 0.49  (-0.09 to 1.07)  p=0.10 |
| log(base2) ART coverage | -1.74  (-4.83 to 1.36)  p=0.27 | -- | -- | -1.60  (-4.28 to 1.07)  p=0.24 |

* β represent changes in mean systolic blood pressure (mmHg) per unit increase for each co-variable of interest; ^#^ Model 1 values are unadjusted estimates (represent separate statistical models); ^##^ Model2 value is adjusted for age and sex of the study population and year of HIV estimate (in 5 year bands); ^## #^Model 3 and 4 values are adjusted for all co-variables included in the same statistical model in the same column and sex of study population and year of HIV estimate (in 5 year bands); GNI = Gross National Income; ART= Antiretrovial therapy; All models used robust standard errors to allowing for clustering by countries
